# Enhancing radiotherapy outcomes in rectal cancer: A systematic review of targeting hypoxia-induced radioresistance

**DOI:** 10.1016/j.ctro.2023.100695

**Published:** 2023-10-28

**Authors:** Matthew Fok, Rhianna Hill, Hayley Fowler, Rachael Clifford, Aaron Kler, Jayanma Uzzi-Daniel, Sonia Rocha, Gabrielle Grundy, Jason Parsons, Dale Vimalachandran

**Affiliations:** a Institute of Systems, Molecular and Integrative Biology University of Liverpool, UK; b Countess of Chester Hospital, Colorectal Surgery Department, Chester, UK; c Institute of Cancer and Genomic Sciences, University of Birmingham, UK

**Keywords:** Rectal cancer, Tumour hypoxia, Radiotherapy, Hypoxia

## Abstract

**Introduction:**

Neoadjuvant radiotherapy is successfully used in rectal cancer to improve overall survival. However, treatment response is both unpredictable and variable. There is strong evidence to show that the phenomenon of tumour hypoxia is associated with radioresistance, however the mechanism(s) behind this are poorly understood. Consequently, there have only been a small number of studies evaluating methods targeting hypoxia-induced radioresistance. The purpose of this systematic review is to evaluate the potential effectiveness of targeting hypoxia-induced radioresistance in rectal cancer and provide recommendations for future research in this area.

**Methods:**

A comprehensive literature search was performed following the PRISMA guidelines. This study was registered on the Prospero database (CRD42023441983).

**Results:**

Eight articles met the inclusion criteria. All studies identified were *in vitro* or *in vivo* studies, there were no clinical trials. Of the 8 studies identified, 5 assessed the efficacy of drugs which directly or indirectly targeted hypoxia and three that identified potential targets. There was conflicting *in vivo* evidence for the use of metformin to overcome hypoxia induced radioresistance. Vorinostat, atovaquone, and evofosfamide showed promising preclinical evidence that they can overcome hypoxia-induced radioresistance.

**Discussion:**

The importance of investigating hypoxia-induced radioresistance in rectal cancer is crucial. However, to date, only a small number of preclinical studies exist evaluating this phenomenon. This systematic review highlights the importance of further research to fully understand the mechanism behind this radioresistance. There are promising targets identified in this systematic review however, substantially more pre-clinical and clinical research as a priority for future research is needed.

## Highlights

• Hypoxia induced radioresistance is an under researched area in rectal cancer.
• There are several preclinical trials which show promise in sensitizing hypoxic cells to radiation.
• The ability to manipulate the hypoxic response of cancer cells can potentially de-escalate radiotherapy and improve outcomes.
• More research is needed in hypoxia-induced radioresistance in rectal cancer.

## Introduction

A diagnosis of rectal cancer is devastating, and patients are often subjected to a challenging regime of chemoradiotherapy followed by major resectional surgery. Neoadjuvant chemoradiotherapy has been successfully used to downstage the disease, reduce local recurrence rates, improve survival, and, more recently, induce a complete clinical and/or pathological response [1]. Despite significant advances in radiotherapy, the response is both unpredictable and variable, with approximately 5–30 % of patients experiencing disease progression whilst undergoing chemoradiotherapy [2], [3]. Furthermore, a significant number of patients (∼80 %) will experience adverse side effects and toxicity whilst undergoing radiotherapy, including diarrhoea, incontinence, fistulas, sexual dysfunction and pain [4]. Cumulatively, these demonstrate some of the challenges and uncertainties associated with this treatment.

There is strong evidence to show that the phenomenon of tumour hypoxia, particularly in solid tumours, is associated with radioresistance [5], [6], [7]. Tumour hypoxia is defined as a reduced oxygen availability and arises from the complex interplay of multiple factors, including inadequate blood perfusion, uncontrolled rapid tumour proliferation, and elevated metabolic needs, which collectively culminate in an unavoidable oxygen-deficient environment. It is generally accepted that the oxygen level in hypoxic rectal cancer tumour tissues averages between 1 % and 2% O_2_ or below [8]. Genome-wide and microRNA analyses have consistently demonstrated that colorectal cancers (CRC) exhibiting a hypoxic tumour microenvironment equate to significantly poorer clinical outcomes, particularly in terms of disease-free survival [9], [10], [11], [12]. Furthermore, there is evidence indicating that radiologically detected hypoxia employing a novel hypoxia marker ^60^Cu-diacetyl-bis (N4-methylthiosemicarbazone), is a predicative factor for diminished survival and reduced tumour response to neoadjuvant chemoradiotherapy in patients diagnosed with rectal cancer [13]. There are several prevailing theories as to why tumour hypoxia leads to radioresistance. The oxygen fixation hypothesis suggests that the DNA damage caused as a consequence of the free radicals generated in response to radiotherapy, is more difficult to repair when caused by the product of a radical and an oxygen molecule [14]. Conversely, the absence of oxygen means that DNA damage is not fixed and therefore can undergo repair. However, more recently research is being developed around the intrinsic cellular adaptations to hypoxia and within the tumour microenvironment. This can lead to alterations in gene expression, increased cellular signalling, and activation of survival pathways, all of which can contribute to radioresistance [15]. The hypoxia induced factors (HIFs) are a group of transcriptional factors that function as master regulators of oxygen homeostasis [16]. Activation of HIF in response to hypoxia in tumour cells has been associated with disruption in the ability of DNA repair, the inhibition of apoptosis, and alterations of the cellular metabolism [17]. However, due the complexity of the HIF signalling pathway in response to various concentrations of oxygen further research is needed to fully elucidate the mechanisms underlying the role of HIF activation in promoting radioresistance in specific tumours under hypoxic conditions. Furthermore, research into hypoxia has revealed that many HIF-independent mechanism are activated in hypoxia, including chromatin reprogramming [18], [19], inflammation [20] and control of cell cycle [21]. All of these mechanisms have the potential to contribute to radioresistance but have not been tested *in vitro* or *in vivo*.

The purpose of this systematic review is to evaluate the potential effectiveness of targeting hypoxia-induced radioresistance in rectal cancer and provide recommendations for future research in this area.

## Methods

### Literature search strategy

**Fig. 1:**
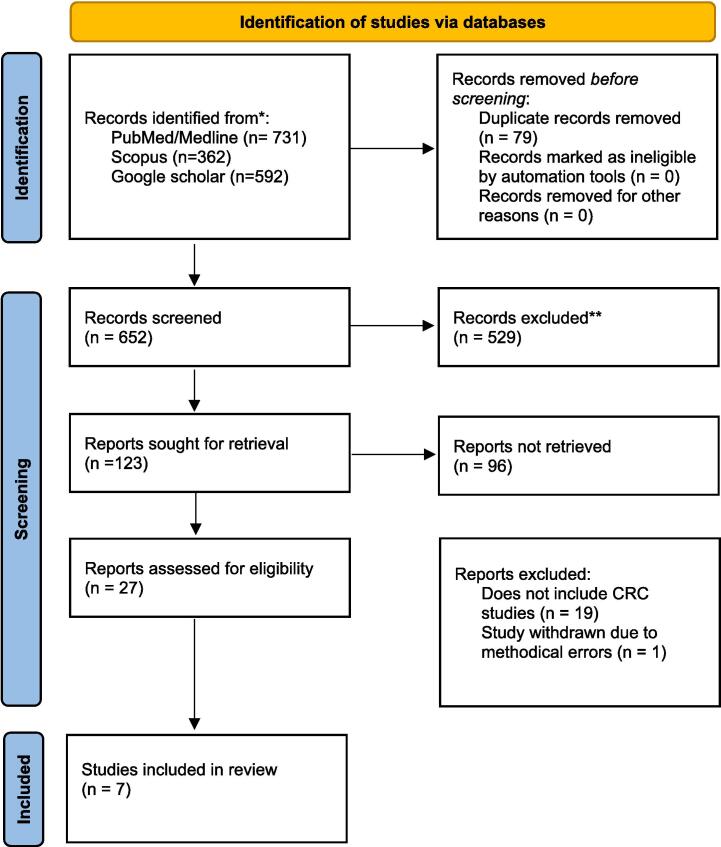
Prisma flow chart.

A comprehensive literature search was conducted according to the Preferred Reporting Items for Systematic Reviews and Meta-Analyses (PRISMA) guidelines. A search was performed on PubMed/Medline, Scopus, and Google scholar from inception to March 2023. Search terms included; “colorectal neoplasms”, “colorectal cancer”, “rectal cancer”, “colon cancer”, “radiotherapy”, “radiation therapy”, “radiotherapeutics”, “radiooncology”, “hypoxia”, “tumour hypoxia”, “oxygen deficiency”, “low oxygen tension”, “radioresistance”, ”radiotherapy resistance“, ”radioresistant“, ”resistance to radiation“, ”hypoxia-inducible factor 1”, “HIF-1”, ”hypoxia-inducible factor-1”, “HIF-1alpha”. All search terms were combined with Boolean operators and searched with MeSH terms to ensure maximal sensitivity. After titles and abstracts were screened using the inclusion and exclusion criteria stated below, full-text article reference lists were searched for any further articles that were suitable for inclusion (Fig. 1). The full study protocol was registered on the Prospero database (CRD42023441983).

### Inclusion and exclusion criteria

All study designs that investigate the effectiveness of targeting hypoxia-induced radioresistance in colorectal cancer radiotherapy were included to capture a broad range of evidence on the topic. Exclusion criteria included studies that were not related to the topic of interest, studies that were conducted on other types of cancer, studies that were not available in full-text format, those not available in the English language.

### Quality assessment

Quality assessment was performed with a modified version of the Collaborative Approach to Meta-Analysis and Review of Animal Data from Experimental Studies (CAMARADES) checklist was used to assess the study quality (Table 2) [22], [23].

## Results

Our literature search revealed 652 potential studies after removal of duplicates (Fig. 1). Following screening of title and abstract, 20 papers were excluded leaving 7 potential studies for which the full text was obtained and reviewed. Of these papers, the reference list was searched to look for any potential papers to include. A total of 8 papers met the inclusion criteria. All studies were *in vitro* or mouse *in vivo* studies. There were no clinical trials identified. Of the 8 studies identified, 5 assessed the efficacy of drugs which directly or indirectly targeted hypoxia and three identified potential mechanistic targets (Table 1).

**Table 1:** Study characteristics.

Author	Year	Type of study	Cell lines used	Patients samples	Drug tested/Target identified	Major Findings
de Mey	2019	*in vitro/in vivo*	HCT116, DLD-1, HT29, SW480, and CT26	no	Metformin and phenformin	Metformin improved the hypoxic radiosensitivity of CRC cells with enhancement ratios of 1.72- and 2.86-fold. CT26 tumour-bearing mice treated with metformin or phenformin with radiation substantially delayed tumour growth with a 1.3 and 1.5 fold enhancement
De Bruycker	2019	*in vivo*	colo205-tumours	no	Metformin	No difference in tumour doubling time between colo205 tumour bearing mice that received radiation with control or metformin
Saelen	2012	*in vitro/in vivo*	HCT116, HT29, and SW620, and KM20L2	no	Vorinostat	Tumour growth delay in mice treated with a combination of ionising radiation and Vorinostat was significantly higher compared to control
Ashton	2016	*in vitro* / spheroids	HCT116	no	Atovaquone, metformin and phenformin	Spheroids treated with metformin and phenformin showed reduction of hypoxia with EF5 immunostaining but not to the extent of atovaquone. Xenografts showed that treatment with atovaquone was able to abolish hypoxia.
Haynes	2018	*in vivo*	Patient-derived colorectal cancer xenografts	no	Evofosfamide	Evofosfamide increased the number of γH2AX foci in xenografts treated with either, 5-FU, radiation or a combination of both
Classen	2019	*in vitro*	HCT116, HEK293T	no	Autophagy	Inhibition of autophagy does not generally increase radiotherapy success and may also lead to an unfavourable outcomex especially under amino acid and oxygen restriction.
Sun	2014	*in vitro*	SW480, SW620	no	Autophagy	Under hypoxia, HIF-1α induces miRNA-210 which in turn enhances autophagy and reduces radiosensitivity by downregulating Bcl-2 expression
Kawai	2016	*in vitro* 2d and clinical tissue samples	HCT116 LoVo HT29 SW480 DLD-1 KM12SM	222 samples	ALODA	*In vitro*, ALDOA expression was negatively associated with chemo- and radiosensitivity and positively associated with proliferation, sphere formation and invasion in both normoxia and hypoxia.

**Table 2:** Quality assessment using CAMARADES checklist.

Num	Criteria	de Mey 2019	de Bruycker 2019	Saelen 2012	Haynes 2018
1	Publication in peer-reviewed journal	Y	Y	Y	Y
2	Statement of control of temperature	NM	Y	NM	NM
3	Randomization of treatment or control	Y	Y	Y	Y
4	Allocation concealment	NM	NM	NM	NM
5	Blinded assessment of outcome	NM	NM	NM	NM
6	Avoidance of anesthetics with marked intrinsic properties	NM	Y	NM	Y
7	Use of animals with cancer	Y	Y	Y	Y
8	Sample size calculation	NM	NM	NM	NM
9	Statement of compliance with regulatory requirements	Y	Y	Y	Y
10	Statement regarding possible conflict of interest	Y	Y	Y	Y
11	Physiological monitoring	NM	Y	NM	Y
12	Prespecified inclusion and exclusion criteria	NM	NM	NM	NM
13	Reporting animals excluded from analysis	NM	NM	Y	NM
14	Reporting of study funding	Y	Y	NM	Y
	*Total score*	6	9	6	8

Abbreviations: Num – number in CAMARADES checklist Y = yes, NM = Not mentioned.

### Drugs

#### Metformin

Three studies assessed the effect of the anti-diabetic biguanide drugs metformin or phenformin to try and overcome hypoxic-induced radioresistance *in vitro* and mouse *in vivo* experiments (Table 1) [24], [25]. De Mey *et al* [26] utilised one murine and four human cell lines (CT26, HCT116, DLD-1, HT29, SW480 respectively) and cultured them in aerobic conditions or subjected them to metabolic hypoxia in a micropellet model (this model cannot give a percentage of oxygen the cells were exposed to). Cells were then dosed with metformin (1–9 µM) or phenformin (10–100 µM) before exposed to radiation (0–9 Gy). The use of both metformin and phenformin significantly improved the radiosensitivity of hypoxic colorectal cancer cells. In this study, phenformin overcame hypoxic radioresistance with enhancement ratios of 1.75 and 2.87 for CT26 and HCT116 tumour cells. Metformin improved the hypoxic radiosensitivity of CT26 and HCT116 with enhancement ratios of 1.72- and 2.86-fold. This was potentially explained through the inhibition of complex 1 activity and impaired oxygen consumption. They further validated their experiments with CT26 tumour-bearing mice. The addition of metformin (300 mg/kg) or phenformin (200 mg/kg) with radiation (9 Gy) substantially delayed tumour growth with a 1.3 and 1.5 fold enhancement, as well as significantly increasing the survival rate.

De Bruycker *et al* [25] studied colo205-bearing mice who received intravenous metformin before radiation. This study exhibited no difference in tumour doubling time between mice that received radiation (15 Gy) with control or metformin (100 mg/kg). The authors scanned the mice with [18F]HX4 hypoxia μPET/computed tomography (CT) scan at baseline and post-metformin dosing. In mice treated with metformin, there was a significant mean intratumoral reduction in [18F]HX4 tumour-to-background ratio compared to saline treated mice. The [18F]HX4 hypoxia μPET technique holds promise for accurately measuring tumour hypoxia. This is because [18F]HX4, when exposed to low oxygen levels, undergoes reduction, and produces reactive intermediates that are subsequently accumulated by viable hypoxic cells [27].

These two studies have shown conflicting evidence with regards to metformin as a radiosensitiser, De Mey *et al* showed that in several CRC cell lines they were able to achieve significant radiosensitisation with metformin [24]. This was further confirmed *in vivo* with CT26 tumour-bearing mice. Conversely, De Bruycker *et al* used Colo205-bearing mice but found metformin made no difference in the radiosensitivity of the tumours [25]. Ashton et al also evaluated metformin and radiotherapy in 3D HCT-116 spheroids, however, except for showing that hypoxia was relieved on admission of metformin they did not report how it effects spheroid growth.

#### Histone deacetylase inhibition

There is increasing evidence that histone deacetylase inhibition (HDACi) lower the cell's capacity to repair IR-induced DNA damage, both at the level of damage signalling and by affecting the major DNA repair pathways (NHEJ and HR), in many different cell types *in vitro* [28]. Saelen *et al* [29] investigated reversing the radioresistant hypoxic phenotype *in vitro* and *in vivo* with the pan-HDACi vorinostat. The authors used four different colorectal cancer cell lines (HCT116, HT29, and SW620 and KM20L2) and athymic Balb/c mice with colorectal cancer xenografts. Vorinostat (1 or 2 μM) was able to reverse the hypoxia-induced radioresistance (0 – 5 Gy) in hypoxia-cultured cells (1 % O_2_). The authors report the results in surviving fractioning (SF) of cells following clonogenic assay (HT29, SF 0.24 ± 0.02 versus 0.51 ± 0.05; p = 0.005; SW620, SF 0.052 ± 0.03 versus 0.36 ± 0.10; p = 0.04; KM20L2, SF 0.040 ± 0.006 versus 0.20 ± 0.02; p = 0.002). *In vivo* studies compared tumour growth delay at 2-fold increase of relative tumour volume (TGD2x) in mice treated with a combination of ionising radiation and vorinostat. TGD2x was significantly higher in mice with hypoxic tumours treated with ionising radiation 6.07 ± 2.5 versus hypoxic tumours treated with ionising radiation and vorinostat − 1.43 ± 3.6; p = 0.015. In this study hypoxia in xenografts was achieved by clamping the tumour during and before irradiation for a total of five minutes. Radiation exposure inhibited the growth of normoxic SW620 xenografts but not of hypoxic tumours. Vorinostat was able to significantly enhance the radiosensitivity of hypoxic xenografts.

#### Atovaquone

Atovaquone, a naphthoquione with broad spectrum antiprotozoal activity, was investigated by Ashton *et al* [30] in HCT116 colorectal cancer spheroids. In an avascular 3D tumour environment, atovaquone was able to alleviate spheroid hypoxia. Immunostaining of athymic BALB/c nude female mice HCT116 xenografts showed that treatment with atovaquone was able to abolish hypoxia. The authors also assessed the effects of metformin (2 mM) and phenformin (10 μM) on spheroid hypoxia with EF5 immunostaining and found that they were able to significantly reduce hypoxia but not to the extent of atovaquone. Spheroid growth was not assessed post-irradiation in the HCT116 model.

#### Evofosfamide

Haynes et al [31] investigated the hypoxia-activated prodrug evofosfamide with patient-derived colorectal cancer xenografts and spheroids. Cells were cultured in 2 % O_2_ before being treated with a combination of 5-fluorouracil (5-FU), evofosfamide and radiation. Under hypoxic conditions, the number of colorectal cancer-initiating cells increased, exhibiting a canonical trait of heightened capacity for self-renewal. The addition of evofosfamide was able to significantly increase the radiosensitivity of spheroids and patient-derived colorectal cancer xenografts as well as decreasing the fraction of colorectal cancer-initiating cells. This was demonstrated with immunofluorescent staining to γH2AX of spheroids. Treatment with 5-FU or radiation in combination with evofosfamide resulted in a further increase in the proportion of cells with γH2AX foci (97 % for 5-FU + evofosfamide or 91 % for radiation + evofosfamide). This included a 2.0- to 5.2-fold increase in the proportion of cells with > 50 foci compared with either agent given alone. Finally, the authors tested [18F]-FAZA-PET/CT imaging to assess for tumour hypoxia in mice with implanted patient-derived colorectal cancer xenografts. In those with high uptake of [18F]-FAZA (increased intratumoral hypoxia), evofosfamide had the greatest effect on tumour growth rate.

### Targets

#### Autophagy

Autophagy is a highly conserved cellular process that involves the degradation and recycling of intracellular components through the formation of double-membrane vesicles, known as autophagosomes [32]. Two papers have assessed how manipulating autophagy affects radiosensitivity in colorectal cancer (Table 1). Classen *et al* [33] described culturing HCT116 colorectal cancer cells *in vitro* under 1 % O_2_ hypoxic conditions. The authors impaired autophagic flux by and siRNA knock down of two key proteins, ATG7 and Beclin 1, and autophagic flux was further impaired with glutamine starvation. However, under hypoxic conditions radiosensitivity was unchanged with Beclin 1 gene knockdown, and furthermore, survival increased with an ATG7 targeted knockdown. The authors concluded that these results highlight that inhibition of autophagy does not generally increase radiotherapy success and may also lead to an unfavourable outcome especially under amino acid and oxygen restriction.

Sun *et al* [34] experimented with colon cancer cell lines SW480 and SW620 under 2 % O_2_ hypoxic conditions. The authors silenced HIF-1α to manipulate miR-210, which promotes migration and invasion, and highly expressed under hypoxic conditions. The inhibition of HIF-1α decreased miR-210 expression and autophagy, furthermore siRNA-mediated silencing of miR-210 upregulated Bcl-2 expression. Autophagy-related proteins including Beclin-1, ATG12 and LC3II as well as the ratio of LC3II/LC3I were decreased after miRNA-210 siRNA treatment under hypoxic treatment compared with hypoxic treatment only group. The authors conclude that upregulation of Bcl-2 reduced the survival fraction of colon cancer cells after radiation treatment (4 Gy) which was not related to apoptosis. However, the results underpinning this conclusion are difficult to interpret. They report that under hypoxic conditions, apoptosis induced by X-ray irradiation (RT) increased after miR-210 siRNA transfections, compared with RT alone. The rate of apoptosis did not differ when cells were co-transfected with miRNA-210 and Bcl-2 siRNA. The authors suggest that the inhibition of miR-210 leads to increased radiosensitivity by upregulating Bcl-2 expression under hypoxic conditions (although not back to control, no values are provided). They further conclude that under hypoxia, HIF-1α induces miRNA-210 which in turn enhances autophagy and reduces radiosensitivity by downregulating Bcl-2 expression in colon cancer cells. It should be noted that the relationship between upregulation of Bcl-2 expression and autophagy is complex and can vary depending on cellular context [35], [36].

These two studies assessing how autophagy relates to radiosensitivity in CRC depicts its complexity and heterogeneity [37]. Classen et al used HCT-116 CRC cell line in 1 % O2 which is different to Sun et al who use the SW480 and SW620 cell lines in 2 % O2. This makes comparisons very difficult to make, however there is good evidence that hypoxia drives the promotion of autophagy. The radiobiological response to autophagy activation in hypoxia however is likely to be cell line and tumour dependant and there is still a lot of work required to evaluate how this can translate clinically.

#### Fructose-bisphosphate aldolase A

Other than autophagy, an association between glycolysis targets and hypoxia-induced radioresistance in rectal cancer has been investigated. Here, Kawai *et al* [38] used hypoxic tumour cells of metastatic liver tissue from patients with colorectal cancer (CRC) as an ‘*in vivo’* hypoxia culture model. The authors investigated the expression of fructose-bisphosphate aldolase A (ALDOA), one of the glycolytic enzymes that catalyzes the reversible conversion of fructose-1,6-bisphosphate to glyceraldehyde-3-phosphate and dihydroxyacetone phosphate, which is a downstream target of HIF-1α. They used six colorectal cancer cell lines, HCT116, LoVo, HT29, SW480, DLD-1 and KM12SM and utilised 1 % O_2_ hypoxic conditions. *In vitro*, ALDOA expression was negatively associated with radiosensitivity and positively associated with proliferation, sphere formation and invasion in both normoxia and hypoxia. They validated their findings through univariate and multivariate analyses of microarray data from 222 resected colorectal cancer samples and revealed that ALDOA was an independent prognostic factor.

## Discussion

This systematic review has highlighted the importance of further investigating tumour hypoxia in colorectal cancer, as it remains an unexplored area with significant potential as a target for improving treatment efficacy. The review has identified that targeting hypoxia-induced radioresistance could be a promising approach to enhance radiotherapy outcomes in patients with rectal cancer. Only pre-clinical experimentation currently exists investigating this important area. One possible explanation for this is given by Fletcher *et al* who described that the majority of studies assessing hypoxia and radiotherapy were performed at the end of the twentieth century when radiotherapy for rectal cancer was not routinely employed [39]. To bridge the gap between pre-clinical experimentation and clinical translation, further understanding of the key molecular mechanisms underlying hypoxia-induced radioresistance is needed.

This review identified four drugs which have been used to overcome radioresistance caused by hypoxia. Metformin and phenformin, two widely used anti-diabetic biguanide drugs, have been recently explored for their potential anti-cancer effects [40]. However, there is ongoing debate regarding their mechanisms of action and efficacy as cancer therapies. Within the context of this review, it was found that metformin can modulate intra-tumoural hypoxia, a key driver of tumour progression and treatment resistance. However, the studies that assessed the impact of metformin on cell survival in cancer cells produced conflicting results indicating the need for further research to better understand its potential anti-cancer effects. Whilst the modulation of hypoxia by metformin is an interesting finding, more studies are needed to elucidate the specific mechanisms of action and the potential clinical utility of these drugs as cancer therapies.

There is accumulating preclinical evidence that HDACi can radiosensitise cancer cells both *in vitro* and *in vivo* [28], [41]. Changes on chromatin structure are expected to affect radiosensitivity. HDACi facilitates chromatin relaxation by increasing the levels of histone acetylation, nevertheless this relaxed structure is not more susceptible to DNA Double Strand Breaks (DSBs) [42]. However, HDACi effectively maintains histones in a hyperacetylated state and hinders the natural process of chromatin refolding into a more condensed structure during repair [43]. There is evidence that elevated histone acetylation levels in the vicinity of DSBs are subsequently replaced by histone post-translational modifications (PTMs) associated with a more condensed chromatin structure. Consequently, HDACi impede these rapid epigenetic transitions, resulting in an altered chromatin state and heightened sensitivity to radiation. This is further compounded by a relationship of HDACis with DNA repair efficacy through the downregulation of key DNA repair proteins such as Ku70, Ku86, Rad51 and DNA-PKc [44]. Finally, a complex relationship exists between, chromatin and its structure in hypoxia, and how this is affected with HDACi, DNA repair and radiotherapy [45], [46]. For example, Vorinostat, a pan-HDACi, commonly used in resistant or recurrent cutaneous T cell lymphoma has showed promise in overcoming the hypoxic-induced radioresistance *in vitro* and *in vivo* experiments [29], [47]. There is only one study assessing the impact of Vorinostat, on hypoxia induced radiosensitivity in colorectal cancer and showed its ability to significantly enhance the radiosensitivity of hypoxic xenografts [29]. More recent literature has shown its potential in altering intratumoural hypoxia signalling not solely attributed to changes in chromatin structure [47].

Evofosfamide is a hypoxia activated prodrug, that in the presence of hypoxia, is reduced to release a cytotoxic bromo-isophosphoramide moiety. It has been tested and shown promise in a variety of different cancers including sarcoma, advanced solids tumours, leukaemia and multiple myeloma as a single agent and not with radiotherapy [48]. Atovaquone is a naphthoquione with broad spectrum antiprotozoal activity [49]. It is used currently as an antimicrobial medication for the prevention and treatment of *Pneumocystis jirovecii* pneumonia, malaria and babesiosis. Its mechanism of action for the treatment of infectious diseases is largely unknown, but is thought to be related to the inhibition of the mitochondrial electron transport chain leading to cell death [49]. However, emerging evidence suggests that it has many other targeted effects which may make it an attractive candidate for cancer treatment [50].

Hypoxia-inducible factor 1 (HIF-1) is a heterodimeric transcription factor composed of an oxygen-sensitive alpha subunit (HIF-1α) and a constitutively expressed beta subunit (HIF-1β). In response to low oxygen levels (hypoxia), HIF-1α protein levels increase and the HIF-1α/HIF-1β complex binds to hypoxia response elements (HREs) in the promoter regions of multiple target genes to activate their transcription [51]. Despite extensive research, a comprehensive understanding of the interplay between hypoxia-inducible factor (HIF) signalling and radiotherapy in the tumour microenvironment is still elusive [16]. This is due in part to the intricate complexity of both the upstream and downstream mechanisms involved in HIF signalling within the tumour microenvironment. This is reflected in a study assessing 86 biopsies of patients with rectal cancer before they underwent long course chemoradiotherapy [52]. Immunohistological staining for HIF-1α and GLUT-1 expression showed no predictive impact regarding response to chemoradiotherapy measured by tumour regression grade (TRG) and was not associated with overall survival. However, despite Kawai *et al* identifying ALODA (a downstream target of HIF-1α) as an independent prognostic factor for colorectal cancer, it is unlikely that a single target would improve hypoxia-induced radiosensitivity in patients with rectal cancer [38]. This is because the molecular mechanisms that contribute to hypoxia-induced radioresistance are complex and multifactorial, involving multiple signalling pathways and cellular processes.

There is an interest in autophagy and its interplay with HIF, which has been shown to play a critical role in cancer cell survival and adaptation to hypoxic conditions [53]. There is debate as to its role in response to radiotherapy as autophagy can promote cell survival and proliferation by recycling nutrients and eliminating damaged organelles or conversely lead to the destruction of essential cellular components and the initiation of cell death pathways [54]. Despite the papers reviewed in this study, it is unclear whether targeting autophagy combined with radiotherapy promotes cell survival or leads to cell death. Further research is needed to fully understand the mechanisms involved in the interplay between autophagy and radiotherapy and to determine the optimal approach to targeting autophagy for therapeutic benefit.

Anti-angiogenic therapies such as bevacizumab, an anti-VEGF monoclonal antibody have been prescribed second line in metastatic colorectal cancer. These drugs have the ability to modify tumour hypoxia. Despite early initial success in clinical trials, prolonged treatment led to resistance to anti-VEGF therapy and cessation of therapy not only led to tumour regrowth but with a more aggressive phenotype [55]. One possible explanation for this is that prolonged anti-VEGF therapy induces intra-tumoural hypoxia and in turn the surviving cells are extremely resistant to therapy. This has been seen in breast cancer xenografts and pancreatic islet tumours [56]. Despite a growing interest in investigating the potential synergistic effects of combining anti-VEGF therapy with radiotherapy there have not been any studies assessing anti-VEGF and radiotherapy [39], [56]. The only clinical trials assessing hypoxic modification in radiotherapy are on-going in head and neck cancer utilising nimorazole as a hypoxic radiosensitiser [57]. The Danish Head and Neck Cancer Study Group (DAHANCA) have been performing several phase I and II trials and have conducted a randomised controlled trial specifically for nimorazole. The results of these trials have shown that nimorazole can significantly enhance the effectiveness of radiotherapeutic management for supraglottic and pharynx tumours, whilst also being well-tolerated by patients. There are also multiple novel hypoxic radiosensitisers in development [58]. There are many significant challenges that need to be addressed, including the considerable heterogeneity of intra-tumoral hypoxia, which makes it difficult to determine which patients will benefit the most from a hypoxic radiosensitizer. Nonetheless, these ongoing studies offer hope for improving the treatment outcomes for head and neck cancer patients, and in the near future, targeting hypoxia in radiotherapy will begin to be trialled in other cancers [59].

### Future perspectives and conclusions

There is an obvious scarcity of knowledge with regards to hypoxia-induced radioresistance in rectal cancer where radiotherapy is commonly employed. However, there are some compelling potential drugs and specific pathways which deserve attention and further research. The are several avenues in which this can be explored further.

o Development of novel hypoxia-activated prodrugs (HAPs) that can specifically target hypoxic tumours and either increase tumour reoxygenation or target intrinsic cellular mechanisms making tumours more sensitive to radiotherapy. An example of a novel HAPs in development includes CP-506 [60].
o Repurposing of existing drugs that show potential in targeting hypoxia-induced radioresistance which have been identified above.
o Understanding the response of the tumour and its microenvironment to hypoxia and radiation to target specific pathways to increase its radiosensitivity.
o The type of radiation delivered to a tumour may potentially alter a hypoxic tumours response. Proton beam therapy has a better relative biological effectiveness (RBE) than photon radiotherapy in both normoxia and hypoxia [61]. Furthermore, it can specifically target hypoxic areas with minimal damage to normal surrounding tissues [62], [63]. Other particle ions (such as carbon ion therapy) may also hold promise considering their higher RBE and lower oxygen enhancement ratio compared to both photons and protons[64].

Overall, investigating the role of hypoxia in colorectal cancer has the potential to significantly improve treatment outcomes for patients and should be a priority area for future research.

Funding: This research did not receive any specific grant from funding agencies in the public, commercial, or not-for-profit sectors.

### CRediT authorship contribution statement

**Matthew Fok:** Conceptualization, Methodology, Formal analysis, Investigation, Data curation, Writing – original draft. **Rhianna Hill:** Conceptualization, Methodology, Validation, Formal analysis, Investigation, Data curation, Writing – original draft. **Hayley Fowler:** Formal analysis, Investigation, Writing – original draft. **Jayanma Uzzi-Daniel:** Investigation, Data curation, Writing – original draft. **Sonia Rocha:** Writing – review & editing, Supervision. **Gabrielle Grundy:** Writing – review & editing, Supervision. **Jason Parsons:** Writing – review & editing, Supervision. **Dale Vimalachandran:** Writing – review & editing, Supervision.

## Declaration of Competing Interest

The authors declare that they have no known competing financial interests or personal relationships that could have appeared to influence the work reported in this paper.
